# Interferon-γ Promotes Inflammation and Development of T-Cell Lymphoma in HTLV-1 bZIP Factor Transgenic Mice

**DOI:** 10.1371/journal.ppat.1005120

**Published:** 2015-08-21

**Authors:** Yu Mitagami, Jun-ichirou Yasunaga, Haruka Kinosada, Koichi Ohshima, Masao Matsuoka

**Affiliations:** 1 Laboratory of Virus Control, Institute for Virus Research, Kyoto University, Sakyo-ku, Kyoto, Japan; 2 Department of Pathology, School of Medicine, Kurume University, Kurume, Japan; National Institutes of Health, UNITED STATES

## Abstract

Human T-cell leukemia virus type 1 (HTLV-1) is an etiological agent of several inflammatory diseases and a T-cell malignancy, adult T-cell leukemia (ATL). HTLV-1 bZIP factor (HBZ) is the only viral gene that is constitutively expressed in HTLV-1-infected cells, and it has multiple functions on T-cell signaling pathways. HBZ has important roles in HTLV-1-mediated pathogenesis, since HBZ transgenic (HBZ-Tg) mice develop systemic inflammation and T-cell lymphomas, which are similar phenotypes to HTLV-1-associated diseases. We showed previously that in HBZ-Tg mice, HBZ causes unstable Foxp3 expression, leading to an increase in regulatory T cells (Tregs) and the consequent induction of IFN-γ-producing cells, which in turn leads to the development of inflammation in the mice. In this study, we show that the severity of inflammation is correlated with the development of lymphomas in HBZ-Tg mice, suggesting that HBZ-mediated inflammation is closely linked to oncogenesis in CD4^+^ T cells. In addition, we found that IFN-γ-producing cells enhance HBZ-mediated inflammation, since knocking out IFN-γ significantly reduced the incidence of dermatitis as well as lymphoma. Recent studies show the critical roles of the intestinal microbiota in the development of Tregs in vivo. We found that even germ-free HBZ-Tg mice still had an increased number of Tregs and IFN-γ-producing cells, and developed dermatitis, indicating that an intrinsic activity of HBZ evokes aberrant T-cell differentiation and consequently causes inflammation. These results show that immunomodulation by HBZ is implicated in both inflammation and oncogenesis, and suggest a causal connection between HTLV-1-associated inflammation and ATL.

## Author Summary

HTLV-1 is a retrovirus which causes a cancer, ATL, and inflammatory diseases of several tissues, such as the spinal cord, eye, skin, and lung. Although these HTLV-1-mediated malignant and inflammatory diseases are recognized as distinct pathological entities, an increased number of HTLV-1 infected cells and enhanced migration/infiltration of infected cells into the lesions are common features of these diseases. Indeed, several clinical observations have suggested a causal link between inflammation and ATL (see Discussion). In order to investigate this issue, appropriate animal models are indispensable. Among HTLV-1-encoded regulatory/accessory proteins, HTLV-1 bZIP factor (HBZ) is thought to be critical to HTLV-1-mediated pathogenesis. We previously reported that HBZ transgenic (HBZ-Tg) mice which express HBZ in CD4^+^ T cells developed both systemic inflammation and T-lymphomas, indicating that they are suitable to evaluate the link, if any, between these phenomena. In this study, we generated several new genetically engineered strains by modifying HBZ-Tg mice, and found that IFN-γ is an accelerator of HBZ-induced inflammation. Importantly, we show that the incidence of inflammation is correlated with that of lymphomagenesis in HBZ-Tg. These findings indicate that modification of T-cell machinery by HBZ is closely associated with both HTLV-1-associated inflammatory diseases and ATL.

## Introduction

Human T-cell leukemia virus type 1 (HTLV-1) infects to mainly CD4^+^ T cells [1], and the provirus is known to exist in effector/memory T cell and regulatory T cell (Treg) subsets [2, 3]. HTLV-1 induces clonal expansion of infected cells and consequently causes a malignancy of CD4^+^CD25^+^ T cells, adult T-cell leukemia (ATL) [1]. This virus also gives rise to inflammatory diseases including HTLV-1 associated myelopathy/tropical spastic paraparesis (HAM/TSP), HTLV-1 uveitis (HU), dermatitis, and HTLV-1-associated bronchoalveolitis (HABA)—diseases which are characterized by infiltration of T cells into the lesions [4–7]. In addition, the incidence of several infectious diseases, e.g., infective dermatitis [8] and strongyloidiasis [9], is higher in HTLV-1 carriers than uninfected individuals, suggesting the presence of HTLV-1-mediated cellular immunodeficiency. These findings indicate that HTLV-1 modifies the immunophenotypes of T cells in the host, and these diseases are induced or promoted by aberrant action of infected T cells. Importantly, some clinical observations imply that in HTLV-1-infected subjects, inflammation accelerates ATL development [10, 11], although a molecular basis connecting inflammation to leukemogenesis has not yet been elucidated. In order to understand the causal link between them, suitable animal models are necessary.

The HTLV-1 provirus encodes several regulatory/accessory genes in its pX region [12]. Among them, *tax* and *HTLV-1 bZIP factor* (*HBZ*), which are encoded in the plus- and minus-strand of the pX region respectively, are thought to be important in pathogenesis. *HBZ* is the only viral gene that is genetically conserved and constitutively expressed in ATL cells [13], whereas Tax is often inactivated by transcriptional silencing or genetic mutations [14, 15]. Moreover, HBZ-transgenic (HBZ-Tg) mice that express *HBZ* in CD4^+^ T cells develop systemic inflammatory diseases, cellular immunodeficiency, and T-cell lymphomas, suggesting that HBZ plays important roles in HTLV-1-mediated pathogenesis [16, 17]. In HBZ-Tg, the number of CD4^+^CD25^+^ T cells and effector/memory CD4^+^ T cells are increased as same as ATL cases [3]. Considering the similarities between phenotypes of HBZ-Tg mice and the clinical features of HTLV-1-infected individuals, the HBZ-Tg mouse model is useful for investigating the mechanisms of pathogenesis by HTLV-1.

We reported previously that the number of induced Tregs (iTregs) was increased in HBZ-Tg mice through upregulation of Foxp3, which is a master gene of Tregs [18]. On the other hand, expression of Foxp3 in HBZ-expressing iTregs is easily lost, whereupon these cells convert to IFN-γ-producing cells that are called exFoxp3 cells [19]. We hypothesized that the increase in iTregs and the concurrent induction of IFN-γ-producing cells are implicated in HBZ-mediated pathogenesis in vivo.

In this study, we focused on the significance of IFN-γ in HBZ-induced inflammation and lymphoma, and established HBZ-Tg/IFN-γ knock out (KO) mice. The incidence of dermatitis was significantly lower in HBZ-Tg/IFN-γ KO mice than HBZ-Tg mice, and importantly, HBZ-Tg/IFN-γ KO mice developed no T-lymphomas. In addition, since the intestinal microbiota have important roles in the development and proliferation of iTregs [20], we generated germ-free (GF) HBZ-Tg mice to evaluate the impact of the intestinal microbiota on the increase in Tregs. Even in aseptic circumstances, HBZ-Tg mice developed dermatitis and had the same pattern of T-cell immunophenotypes as specific pathogen free (SPF) HBZ-Tg mice, suggesting that HBZ causes inflammation in a cell intrinsic manner. We also found that the severity of dermatitis correlates with the development of lymphoma in HBZ-Tg mice. These results suggest a close link between inflammation and oncogenesis in HBZ-Tg mice, and demonstrate the important role of IFN-γ in the molecular mechanism of HBZ-mediated pathogenesis.

## Results

### IFN-γ is involved in the inflammation and lymphomagenesis caused by HBZ

**Fig 1:**
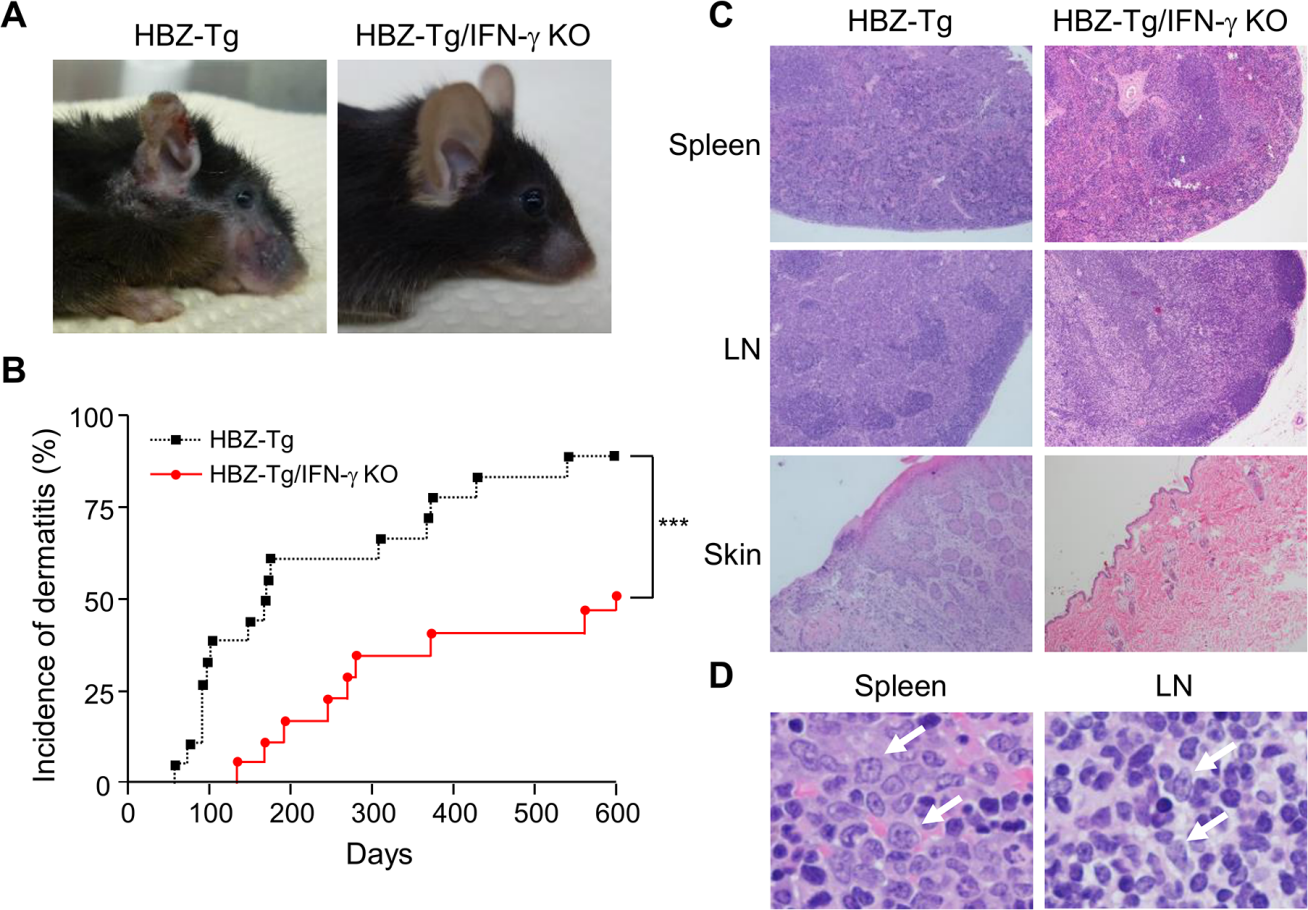
Incidence of inflammation and lymphoma is decreased in HBZ-Tg/IFN-γ KO mice. (A) HBZ-Tg and HBZ-Tg/IFN-γ KO mice at 24 weeks of age. HBZ-Tg mice developed dermatitis around the ears, eyes, and back. HBZ-Tg/IFN-γ KO mice did not develop dermatitis. (B) 90% of HBZ-Tg developed dermatitis by 2 years of age. However, in HBZ-Tg/IFN-γ KO mice, the onset of dermatitis was delayed and its incidence was lower than in HBZ-Tg mice. *** indicates significant differences p<0.01. (C) Histological analysis of HBZ-Tg and HBZ-Tg/IFN-γ KO mice (x10). The spleen of an HBZ-Tg mouse shows a diminution of the structure and a diffuse infiltration of atypical cells in red palps. The lymph node of HBZ-Tg shows a focal diminution of nodal structures and a diffuse infiltration of atypical cells in paracortical area. The skin of HBZ-Tg shows hyperkeratosis of epidermis. However, the spleen, lymph node and skin of HBZ-Tg/IFN-γ KO show normal structures. (D) Lymphomas developed in spleen and lymph nodes of HBZ-Tg mice with dermatitis. In the high magnification (x60), the spleen of an HBZ-Tg mouse shows a diffuse infiltration of atypical large lymphoid cells with irregular nuclei, and the lymph node shows atypical large lymphoid cells, also. Abnormal cells are indicated by white arrows.

In order to analyze the impact of IFN-γ on HBZ-mediated pathogenesis, we crossed HBZ-Tg mice with IFN-γ KO mice to establish HBZ-Tg/IFN-γ KO mice (S1 Fig) [21]. We found that some HBZ-Tg mice developed dermatitis at only 8 weeks of age, and 90% of HBZ-Tg mice developed dermatitis within 2 years (Fig 1A and 1B), and these results are consistent with our previous observations [16]. In contrast, HBZ-Tg/IFN-γ KO mice did not suffer from dermatitis until 19 weeks or older, and after 2 years, only 50% of these mice had developed the skin disease (Fig 1B).

To evaluate the presence of systemic inflammation, we performed histological analysis of multiple organs from ten mice of each genotype at 24 weeks of age. The analysis revealed that 30% of HBZ-Tg mice showed infiltration of lymphocytes into the skin at the time point of analysis, whereas no HBZ-Tg/IFN-γ KO mice showed any abnormalities (Fig 1C and Table 1). Our previous study also showed that HBZ-Tg mice which became moribund had lymphomas [16]. Surprisingly, we found that 30% of HBZ-Tg mice had already developed lymphomas in spleen and lymph nodes at 24 weeks of age—earlier than we had guessed—and more importantly, the severity of inflammation correlated with lymphoma development (Fig 1D and Table 1). In contrast, no HBZ-Tg/IFN-γ KO mice had lymphoma. These data strongly suggest that IFN-γ has an important role in inflammation and lymphoma caused by HBZ, and that inflammation might accelerate oncogenesis in HBZ-expressing T cells.

**Table 1:** Histological findings in WT, HBZ-Tg, IFN-γ KO, and HBZ-Tg/IFN-γ KO mice at 24 weeks of age.

		Dermatitis	Spleen	Skin	LN
WT	#1	-	-	-	-
	#2	-	-	-	-
	#3	-	-	-	-
	#4	-	-	-	-
	#5	-	-	-	-
	#6	-	-	-	-
	#7	-	Congestion	-	-
	#8	-	-	-	-
	#9	-	-	-	-
	#10	-	-	-	-
HBZ-Tg	#1	-	Congestion	-	Atypical lymphocyte
	#2	-	-	-	-
	#3	+++	Lymphoma	++	Lymphoma
	#4	+++	Lymphoma	++	Lymphoma
	#5	++	Lymphoma	+	Lymphoma
	#6	++	Congestion	-	Atypical lymphocyte
	#7	+	-	-	-
	#8	+	-	-	-
	#9	+	-	-	-
	#10	-	-	-	-
IFN-γ KO	#1	-	-	-	-
	#2	-	-	-	-
	#3	-	-	-	-
	#4	-	-	-	-
	#5	-	-	-	-
	#6	-	-	-	-
	#7	-	-	-	-
	#8	-	-	-	-
	#9	-	-	-	-
	#10	-	-	-	-
HBZ-Tg/IFN-γ KO	#1	-	-	-	-
	#2	-	-	-	-
	#3	-	-	-	-
	#4	-	-	-	-
	#5	-	-	-	-
	#6	-	-	-	-
	#7	-	-	-	-
	#8	-	-	-	-
	#9	-	-	-	-
	#10	-	-	-	-

### Foxp3^+^CD4^+^ T cells and effector/memory T cells are increased in both HBZ-Tg and HBZ-Tg/IFN-γ KO mice

**Fig 2:**
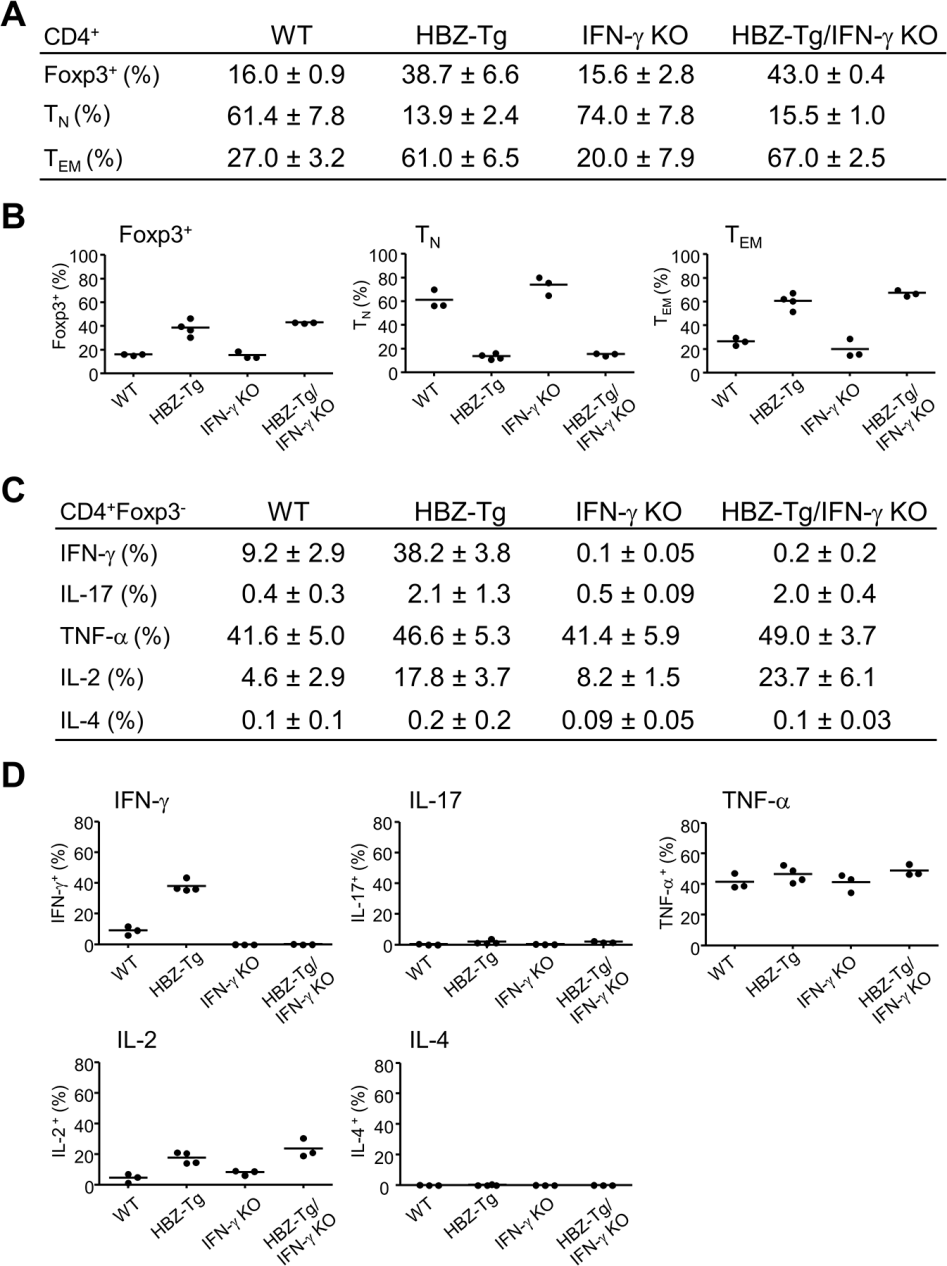
Comparison of T-cell subsets between HBZ-Tg and HBZ-Tg/IFN-γ KO mice. (A and B) Splenocytes were harvested from WT, HBZ-Tg, IFN-γ KO, and HBZ-Tg/IFN-γ KO mice at 24-week old. Cells were stained with anti-CD4, anti-Foxp3, anti-CD25 antibodies for detection of regulatory T cells, and anti-CD44, anti-CD62L antibodies for effector/memory CD4^+^ T cells. Percentages of each subset in CD4^+^ T cells are shown (n = 3 or 4). TN: naïve T cell, TEM: effector/memory T cell. (C and D) Cytokine production in CD4^+^Foxp3^-^ T cells of each strain was evaluated. Splenocytes were stimulated with PMA/ionomycin in the presence of protein transport inhibitor for 4 hours, stained with specific antibodies, and analyzed by flow cytometry. Percentages of each subset in CD4^+^ T cells are shown (n = 3 or 4).

The numbers of Foxp3^+^CD4^+^ T cells and effector/memory T cells are increased in HBZ-Tg [16]. To evaluate the influence of IFN-γ on CD4^+^ T cells, we performed flow cytometry and compared the patterns of T-cell subsets between HBZ-Tg and HBZ-Tg/IFN-γ KO mice. CD4^+^ T cells from HBZ-Tg/IFN-γ KO mice expressed Foxp3 at similar level to that of HBZ-Tg mice (Fig 2A and 2B and S2 Fig). Likewise, the effector/memory population was increased in HBZ-Tg/IFN-γ KO mice (Fig 2A and 2B and S2 Fig), indicating that these changes in CD4^+^ T-cell subset populations in HBZ-Tg mice are independent of IFN-γ production and not directly correlated with the inflammatory phenotypes of the HBZ-Tg mice.

Next, we analyzed the production of inflammatory cytokines. Splenic T cells from 24-week-old mice were stimulated by phorbol myristate acetate (PMA)/ionomycin and the expression of IL-17, TNF-α, IL-2, IL-4 and IFN-γ in CD4^+^ T cells was evaluated by flow cytometry. IFN-γ production was clearly increased in HBZ-Tg mice. Production of IL-17 and IL-2 were also increased in both HBZ-Tg and HBZ-Tg/IFN-γ KO mice (Fig 2C and 2D and S2 Fig). These findings show that loss of IFN-γ does not affect the production of these inflammatory cytokines by HBZ-expressing CD4^+^ T cells.

### Germ-free HBZ-Tg mice didn’t show any phenotypic differences from SPF HBZ-Tg mice

**Fig 3:**
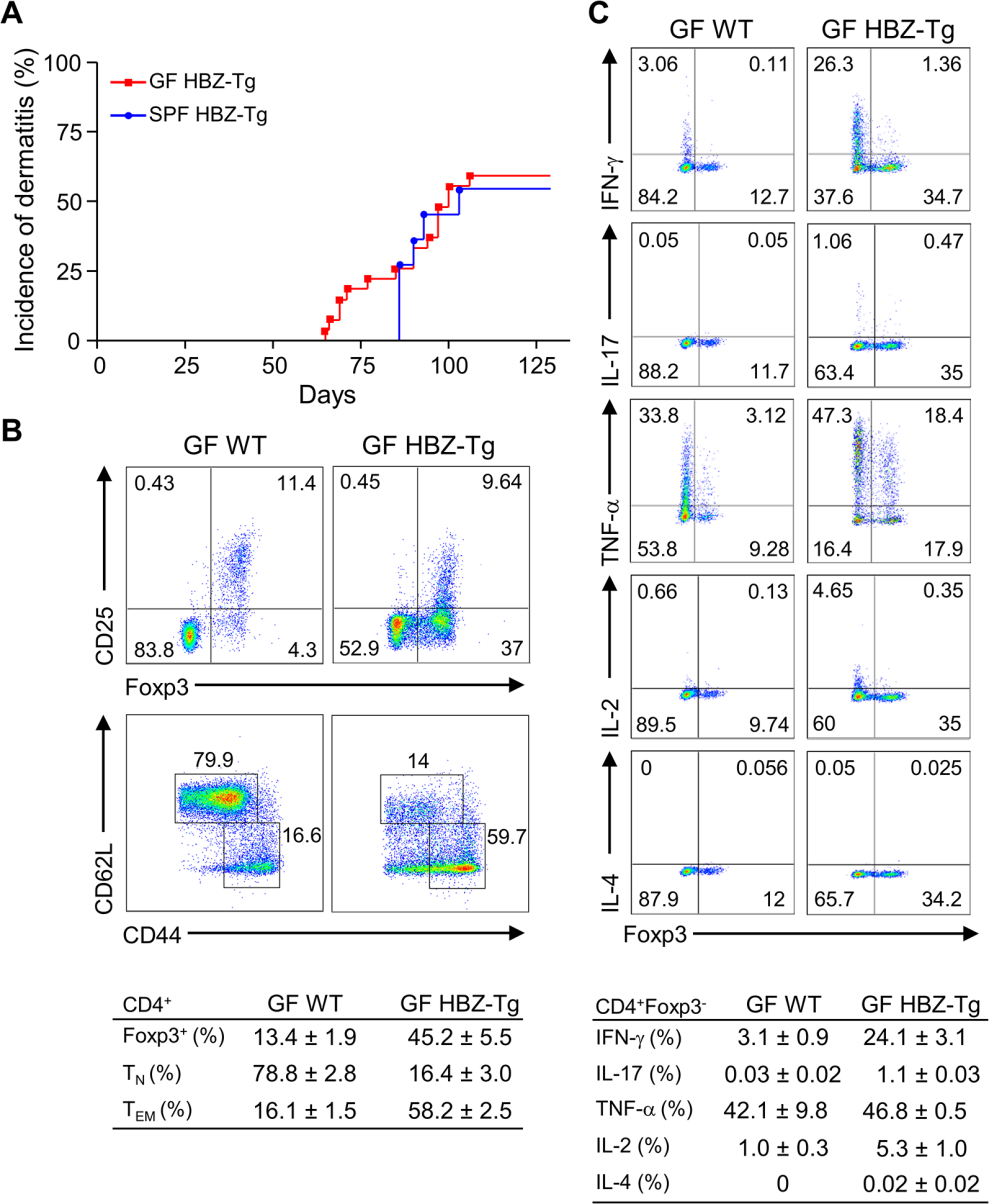
Inflammatory phenotypes of germ-free HBZ-Tg mice. (A) GF HBZ-Tg mice developed dermatitis similarly to SPF HBZ-Tg mice. (B) Splenocytes were harvested from 18-week-old GF HBZ-Tg or GF WT littermates. The percentages of Tregs and effector/memory CD4^+^ T cells were evaluated. Representative results of the dot plots and a summarized table are shown. (C) Cytokine production in CD4^+^ T cells was evaluated. Splenocytes were stimulated with PMA/ionomycin in the presence of protein transport inhibitor for 4 hours, stained with specific antibodies, and analyzed by flow cytometry. Representative results of the dot plots and a summarized table are shown.

Recently, it has been reported that iTregs are most abundant in the colonic mucosa in mice, and that the number of mucosal Tregs is remarkably decreased in germ-free mice, indicating that the gut microbiota has important roles in the development and proliferation of iTregs [20]. Since both HBZ-Tg and HBZ-Tg/IFN-γ KO mice demonstrate increased numbers of iTregs, we asked if the microbiota affected HBZ-mediated iTreg expansion and subsequent inflammation as an extrinsic factor. In order to analyze the impact of microbiota on HBZ-mediated pathogenesis, we generated the germ-free (GF) HBZ-Tg mice, which are genetically the same as the HBZ-Tg mice we reported previously [16]. Contrary to our expectation, these GF HBZ-Tg mice were phenotypically no different than regular HBZ-Tg mice maintained in SPF conditions. The GF HBZ-Tg mice started developing skin inflammation as early as 9 weeks of age, and 16 of 28 (57%) GF HBZ-Tg mice suffered from dermatitis by 18 weeks of age (Fig 3A). Regarding the phenotypes of T cells, there were no significant differences between GF and SPF HBZ-Tg; the number of both effector/memory T cells and Tregs were higher than those in nontransgenic littermates, and the production of IFN-γ was upregulated in HBZ-Tg in both settings (Fig 3B and 3C and S3 Fig). These results imply that the intrinsic activity of HBZ is more important than the intestinal microbiota in influencing the immune modulation, inflammation, and lymphomas observed in HBZ-Tg mice.

### CXCR3 and CXCL10 are not involved in HBZ-mediated inflammation

**Fig 4:**
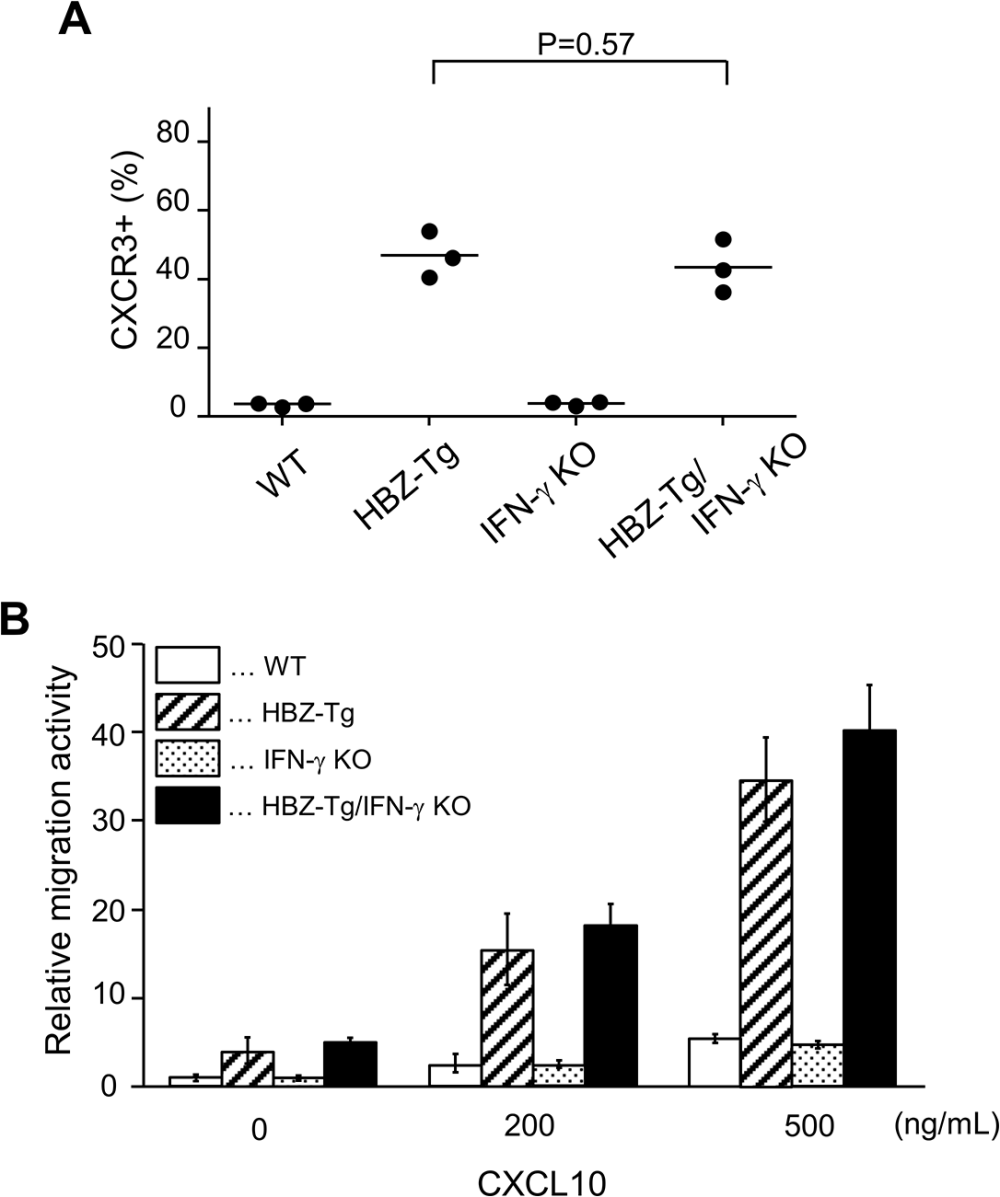
Expression and function of CXCR3 on CD4^+^ T cells in HBZ-Tg and HBZ-Tg/IFN-γ KO mice. (A) Splenocytes were obtained from mice, and the CXCR3 expression level in CD4^+^ T cells was evaluated by flow cytometry. Three mice of each strain were analyzed and the result was summarized in the graph. (B) The migration activity of CD4^+^ T cells towards CXCL10. CD4^+^ T cells were isolated from splenocytes using magnet beads. Murine recombinant CXCL10 was added at concentrations of 0, 200, or 500 ng/mL. Migrating cells were counted by flow cytometry.

In a previous study, we showed that a chemokine receptor, CXCR3, was highly expressed on HBZ-Tg CD4^+^ T cells and that most cells that had migrated into inflammatory lesions were CXCR3 positive [18]. CXCR3 is expressed in IFN-γ-producing Th1 cells [22]. Thus we hypothesized that the reduction of inflammation in HBZ-Tg/IFN-γ KO mice might correlate with reduced CXCR3 expression on their CD4^+^ T cells. We compared CXCR3 expression levels between HBZ-Tg and HBZ-Tg/IFN-γ KO mice, and found that HBZ-Tg/IFN-γ KO mice expressed high levels of CXCR3 on CD4^+^ T cells despite of the absence of IFN-γ (Fig 4A).

Furthermore, we carried out chemotaxis assay to evaluate the function of CXCR3 expressed on CD4^+^ T cells of HBZ-Tg and HBZ-Tg/IFN-γ KO mice. Murine recombinant CXCL10, which is a major ligand of CXCR3, was used as a chemoattractant [22]. CD4^+^ T cells were purified from HBZ-Tg and HBZ-Tg/IFN-γ KO mice, and these cells were placed in the upper chambers. The lower chambers were filled with media containing 200 or 500 ng/mL CXCL10 or control media. The migration capacity of CD4^+^ T cells from HBZ-Tg/IFN-γ KO mice was similar as that from HBZ-Tg mice (Fig 4B). From these results, we conclude that CXCR3 was inducible and functional in HBZ-Tg/IFN-γ KO mice.

**Fig 5:**
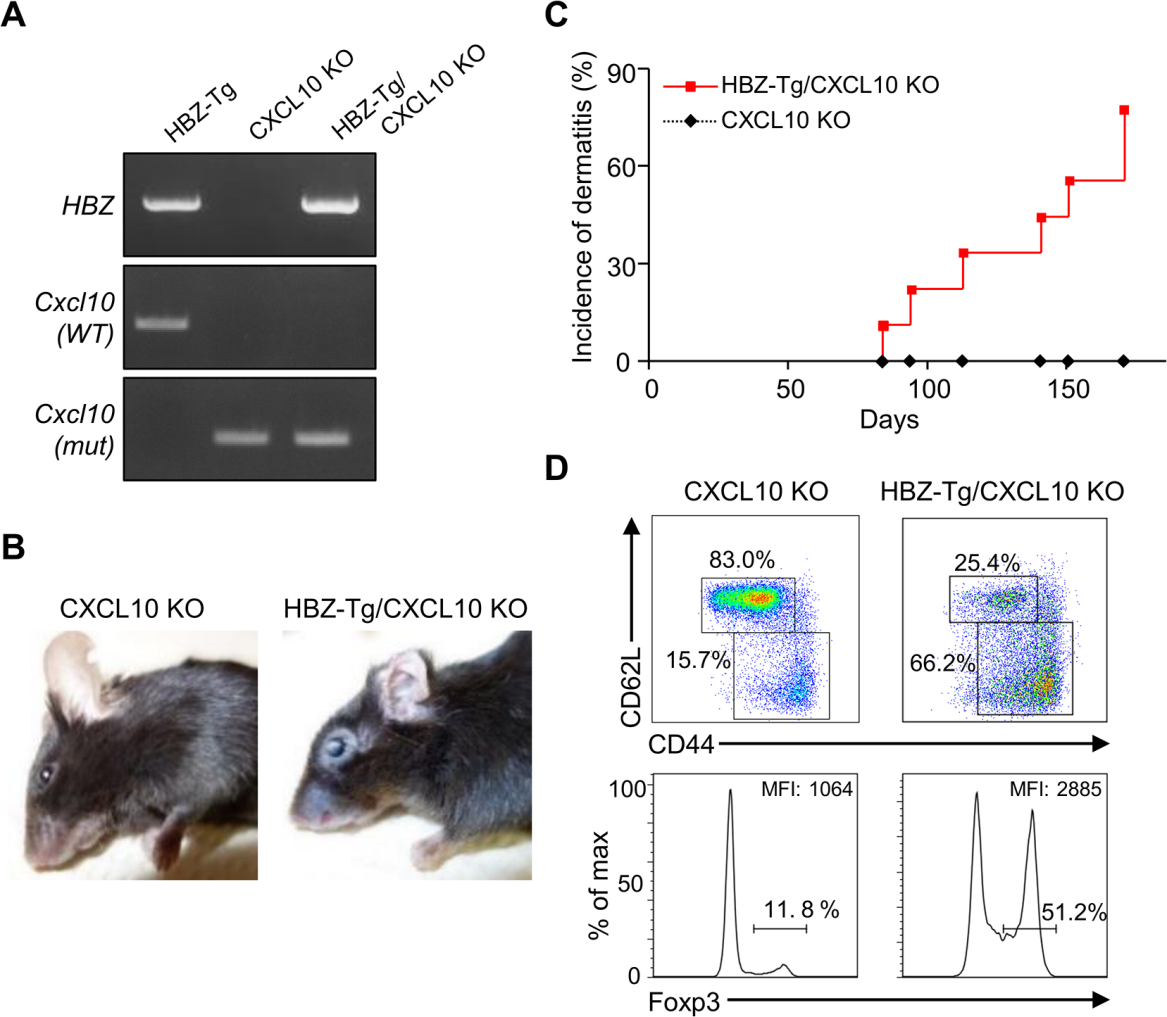
CXCL10 is not associated with systemic inflammation in HBZ-Tg mice. (A) HBZ-Tg and CXCL10 KO mice were crossed to establish HBZ-Tg/CXCL10 KO mice. (B) HBZ-Tg/CXCL10 KO mice developed dermatitis. (C) At 24 weeks old, more than 50% of HBZ-Tg/CXCL10 KO mice developed dermatitis. (D) Splenocytes were obtained from CXCL10 KO mice and HBZ-Tg/CXCL10 KO mice. Cells were stained with anti-CD4, anti-CD44, anti-CD62L, and anti-Foxp3, and analyzed by flow cytometry. Among CD4^+^ T cells of HBZ-Tg/CXCL10 KO mice there were increased numbers of increased effector/memory and Foxp3 expressing cells.

Next, we evaluated the importance of CXCL10 in disease development in HBZ-Tg mice, since CXCL10 is one of the chemokines induced by IFN-γ [23]. To do this, we established HBZ-Tg/CXCL10 KO mice [24] (Fig 5A). HBZ-Tg/CXCL10 KO mice developed dermatitis beginning at 12 weeks old (Fig 5B and 5C). At 24 weeks of age, about 80% of the mice had developed dermatitis (Fig 5C). Histological analysis revealed that HBZ-Tg/CXCL10 KO mice also developed inflammation in several other organs (Table 2). In addition, HBZ-Tg/CXCL10 KO mice showed increases in the numbers of Tregs and effector/memory fraction compared to WT mice (Fig 5D). All phenotypes of HBZ-Tg/CXCL10 KO mice we analyzed were quite similar to those of HBZ-Tg mice. We thus concluded that the CXCR3/CXCL10 axis was not related to pathogenesis in our HBZ-Tg mouse model.

**Table 2:** Histological findings in CXCL10 KO, and HBZ-Tg/CXCL10 KO mice at 24 weeks of age.

		Dermatitis	Spleen	Lung	Skin
CXCL10 KO	#1	-	-	-	-
	#2	-	-	-	-
	#3	-	-	-	-
	#4	-	-	-	-
	#5	-	-	-	-
	#6	-	-	-	-
	#7	-	-	-	-
	#8	-	-	-	-
HBZ-Tg/CXCL10 KO	#1	-	-	-	-
	#2	-	-	-	-
	#3	+	-	+	-
	#4	+	-	+	-
	#5	+	-	-	+
	#6	+++	Atypical lymphocyte	+	+++
	#7	+++	Atypical lymphocyte	+	++
	#8	+++	-	+	+
	#9	++	Atypical lymphocyte	+	+

### Identification of candidate molecules implicated in HBZ and IFN-γ mediated pathogenesis

**Fig 6:**
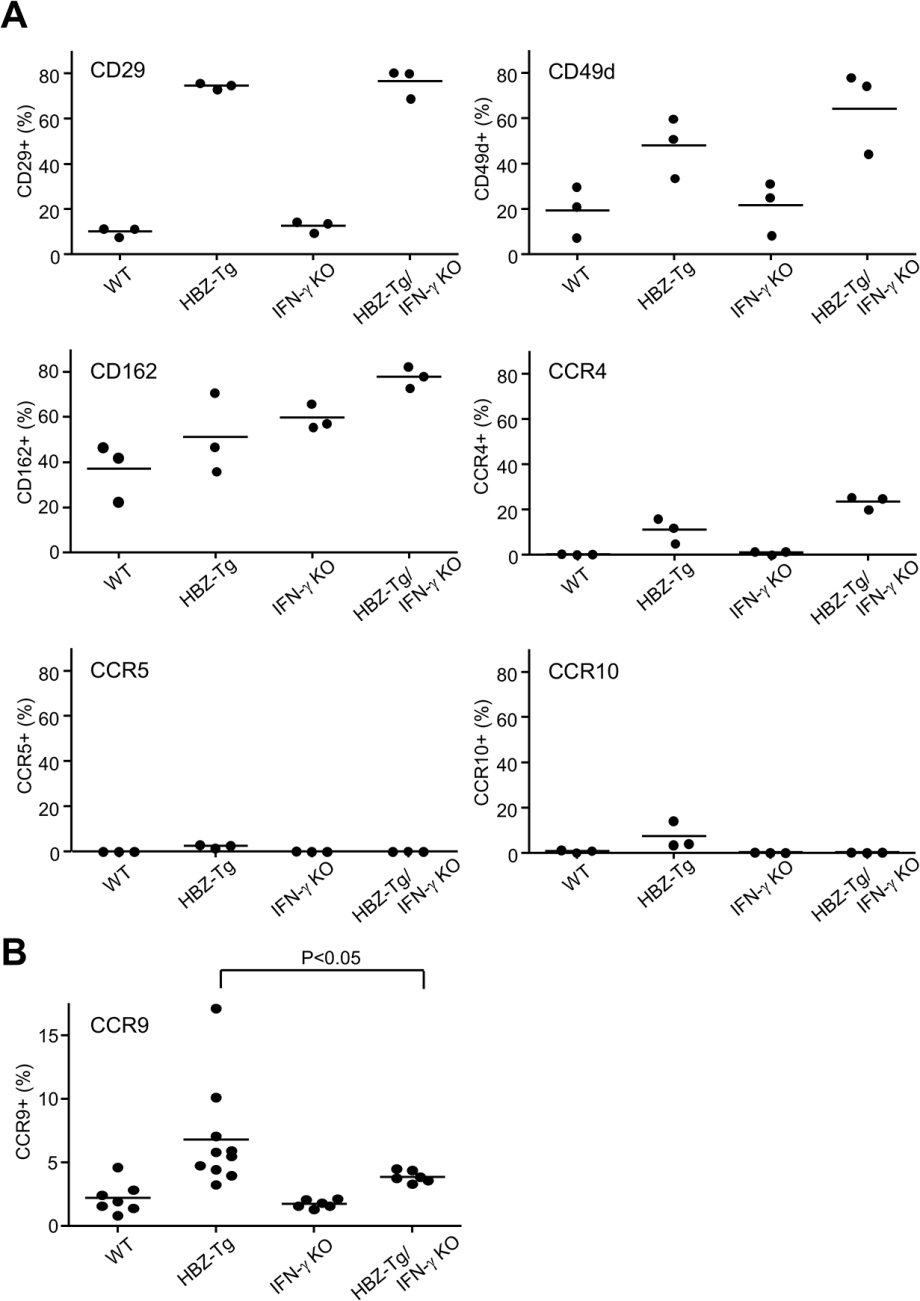
Expression of the chemokine receptors and adherent molecules on CD4^+^ T cells of WT, HBZ-Tg, IFN-γ KO, and HBZ-Tg/IFN-γ KO mice. (A) Splenocytes were stained with antibodies against various chemokine receptors and adhesion molecules. The percentage of CD4^+^ T cells expressing each molecule is shown. Three mice of each strain were analyzed. (B) The expression of CCR9 is significantly upregulated in CD4^+^ T cells of HBZ-Tg but not in those of HBZ-Tg/IFN-γ KO mice.

Although CD4^+^ T cells from HBZ-Tg mice and HBZ-Tg/IFN-γ KO mice were similar in their migratory responses to CXCL10, their abilities to infiltrate tissues in vivo may differ, because the HBZ-Tg/IFN-γ KO mice did not develop dermatitis to the same degree that the HBZ-Tg mice did. Therefore we looked for chemokine receptors or adherent molecules that are highly expressed on T cells in HBZ-Tg but not HBZ-Tg/IFN-γ KO mice. As shown in Fig 6A, most of the molecules studied were highly expressed on CD4^+^ T cells of both HBZ-Tg and HBZ-Tg/IFN-γ KO mice compared with wild type littermates. However, we found that the chemokine receptor CCR9 was upregulated only in HBZ-Tg mice (Fig 6B), suggesting that upregulation of CCR9 is involved in inflammation mediated by HBZ and IFN-γ.

**Fig 7:**
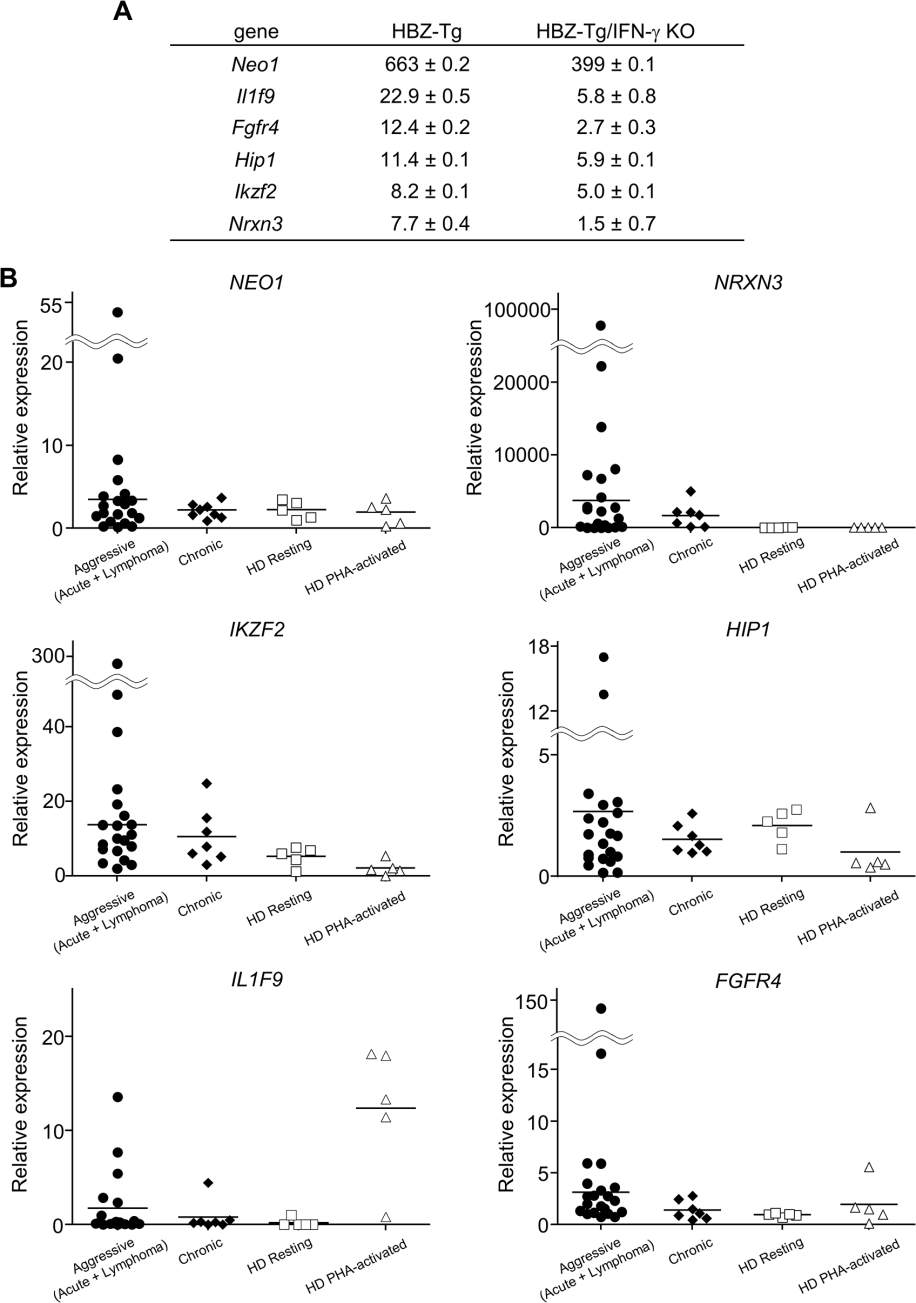
Microarray analysis of WT, HBZ-Tg, IFN-γ KO, and HBZ-Tg/IFN-γ KO mice. CD4^+^ T cells were purified from splenocytes of WT, HBZ-Tg, IFN-γ KO, and HBZ-Tg/IFN-γ KO mice. (A) Validation of the microarray result by qPCR. cDNA of splenocytes from WT, HBZ-Tg, and HBZ-Tg/IFN-γ KO mice were used. Expression levels of the candidate genes were normalized using the values of WT as reference. (B) Transcription levels of the human homologues of the candidate genes in ATL patients and healthy donors (HD). Relative expression values were calculated by the delta delta Ct method using a value of one resting sample as reference. Aggressive: acute and lymphoma types of ATL.

In order to identify further cellular genes implicated in HBZ/IFN-γ-mediated inflammation, we performed DNA microarray analysis. We extracted RNA from CD4^+^ T cells of WT, HBZ-Tg, IFN-γ KO, and HBZ-Tg/IFN-γ KO mice and evaluated the profiles of gene expression. According to the result of microarray, we picked up several genes that were expressed higher in HBZ-Tg than HBZ-Tg/IFN-γ KO, and validated their expression profiles by quantitative RT-PCR. Among these genes, we further looked for the genes that are overexpressed in human ATL cases. Finally, we identified *Neo1*, *Il1f9*, *Fgfr4*, *Hip1*, *Iklf2*, and *Nrxn3* that met these criteria (Fig 7A and 7B). Interestingly, human homologues of these genes were upregulated especially in the aggressive form of ATL (Fig 7B). They are likely to be divided into 2 groups by the pattern of the expression in healthy donor cells. One contains the genes which expression is unchanged or reduced in phytohaemagglutinin (PHA)-stimulated cells compared with resting cells, such as *NEO1*, *NRXN3*, *IKZF2*, and *HIP1*. In contrast, *IL1F9* and *FGFR4* belong to another group in which their transcription are enhanced by PHA, suggesting that they are inducible by potent mitogenic stimulation even in normal T cells. These genes were generally overexpressed in HTLV-1-transformed and ATL cell lines although there were several exceptions (S1 Table). Interestingly, it has been reported that most of them are aberrantly expressed in several types of cancer cells, suggesting that they are associated with the linkage between chronic inflammation and oncogenesis in HTLV-1-infected subjects.

## Discussion

Persistent inflammation is widely recognized as a tumor-promoting factor in many cancers, and it is estimated that about 15% of human malignancies are associated with chronic inflammation and infection [25]. For example, inflammatory bowel diseases, such as ulcerative colitis, are associated with colon cancer [26]. Chronic gastritis caused by *Helicobacter pylori* [27] and chronic hepatitis caused by hepatitis B virus or hepatitis C virus [28] are implicated in development of gastric cancer and hepatocellular carcinoma (HCC), respectively. In these solid tumors, infiltrating immune cells are thought to produce cytokines, chemokines, and growth factors that induce the proliferation of tumor cells [25]. In addition, those inflammatory cells produce reactive oxidative species resulting in genetic instability [29]. Activation of the TNF-α or the NF-κB pathway is important especially in the development of HCC [30] and colon cancer [31].

In the case of HTLV-1 infection, the virus itself dysregulates the functions of CD4^+^ T cells, modifies T-cell subsets, and triggers clonal expansion of infected cells. HTLV-1 causes both inflammation and a malignant disease, but a precise mechanism crosslinking these diseases was not clarified. Several clinical observations have suggested the correlation between HTLV-1-associated inflammatory diseases and ATL. It was reported that the frequency of ATL development in HTLV-1-infected patients with diffuse pan-bronchiolitis was significantly high among all HTLV-1 carriers [10]. In addition, the abundance of certain HTLV-1-infected clones is increased in HTLV-1 carriers with strongyloides and infective dermatitis [11], implying that these inflammatory diseases increase the risk of ATL development. In this study, we found T-cell lymphomas only in HBZ-Tg mice with dermatitis, and severity of inflammation tended to correlate with lymphoma development, suggesting that inflammatory signals induced by HBZ accelerate oncogenic processes. Since there is no immune reaction against HBZ in these mice, HBZ triggers inflammation only by its intrinsic action. This idea is compatible with the findings that, even in a germ-free environment, the number of Tregs was increased in HBZ-Tg mice and they developed systemic inflammation the same as under SPF conditions. These results suggest that the inflammatory phenotypes of HBZ-Tg mice are caused by an inherent function of HBZ, and that HBZ-mediated inflammation promotes oncogenesis in HBZ-expressing CD4^+^ T cells.

In addition, we show here that IFN-γ is an important molecule in the pathogenesis by HBZ. IFN-γ is conventionally recognized as a cytokine that acts in host defense against various pathogens and tumor rejection. IFN-γ is secreted by mainly activated CD4^+^ T cells (Th1 cells), cytotoxic CD8^+^ T lymphocytes, and natural killer cells, and has cytostatic/cytotoxic effects by inducing cell-mediated immune responses [32]. IFN-γ primarily activates the JAK/STAT signaling pathway through interaction with IFN-γR1, and induces the transcription of primary response genes such as IRF family genes. Many of these primary response genes encode transcription factors that induce a lot of secondary response genes to react to the stimulation. Previous studies showed that blockade of IFN-γ/IFN-γR signaling in mice compromised rejection of tumors by the immune system, indicating that IFN-γ functions in immune surveillance against tumors [33–35]. On the other hand, under certain circumstances, IFN-γ is also known to have a protumorigenic function involving proliferative and anti-apoptotic signals in tumor cells [32]. In this study, we found that knocking out of IFN-γ significantly decreased the incidence of inflammation and malignant lymphoma in HBZ-Tg mice, indicating that IFN-γ plays a supportive role in the development of both types of diseases caused by HBZ.

To understand how IFN-γ contributes to HBZ-associated pathogenesis, we looked for cellular factors differentially expressed in CD4^+^ T cells of HBZ-Tg compared with HBZ-Tg/IFN-γ KO mice. These genes are thus implicated in pathogenesis mediated by HBZ and IFN-γ together. CCR9 is an intestine oriented chemokine receptor [36]. This upregulation is consistent with our observation that massive infiltration of lymphocytes was observed in HBZ-Tg mice [18]. We also identified several cancer-related genes which are overexpressed in both HBZ-Tg and ATL patients. *NEO1* encodes a cell surface protein that belongs to the immunoglobulin superfamily. It has been reported that overexpression of *NEO1* in gastric cancer is involved in cell proliferation and migration [37]. *IL1F9*, also known as *IL36gamma*, is an IFN-γ-inducible gene that has been reported to activate NF-κB and MAPK signaling in human T cells [38]. *FGFR4* encodes a member of the fibroblast growth factor receptor family, and implicated in the tumorigenesis of many types of cancers, such as HCC, prostate cancer, breast cancer, pancreatic cancer [39–43]. *IKZF2* encodes a member of the Ikaros family of zinc-finger proteins, Helios, which is mainly expressed in T cell. A recent study showed that aberrant isoforms of *IKZF2* are dominantly expressed in ATL cells, and function in T-cell proliferation and survival [44], suggesting that HBZ might dysregulate the expression pattern of *IKZF2* in ATL cells. *HIP1* is also overexpressed in several cancer tissues like breast cancer and possesses the oncogenic properties through BCL-2 and NF-κB pathways [45]. Taken together, it is possible that HBZ and HBZ-mediated inflammation induce these factors and subsequently trigger transformation in a part of HTLV-1-infected cells. In order to clarify the significance of each factor in HBZ-mediated pathogenesis, further experiments will be required. Interestingly, previous studies on Tax, which is another oncoprotein of HTLV-1, showed that transgenic mice expressing Tax under control of the granzyme B promoter developed LGL leukemia, and knocking out of IFN-γ in this strain enhanced the tumor formation [46, 47], suggesting that IFN-γ has the opposite effect on Tax-mediated oncogenesis that it has on HBZ-mediated oncogenesis. In these Tax-Tg mice, IFN-γ was shown to have an anti-angiogenic effect by suppressing the transcription of VEGF [47]. HBZ and Tax regulate several signaling pathways in opposite manners [1], suggesting that IFN-γ may differentially regulates the effects of HBZ and Tax on HTLV-1-infected cells or HBZ and Tax may regulate IFN-γ in opposite way, in response to the cellular context.

In HAM/TSP patients, IFN-γ-producing cells are increased in a CD4^+^FoxP3^-^ subpopulation, and suggested to have a role in the pathogenesis of this inflammatory disease [48, 49]. A recent study showed that HTLV-1-infected cells in the cerebrospinal fluid expressed IFN-γ and CXCR3, and its ligand CXCL10 was expressed in astrocytes upon stimulation with IFN-γ, leading to an IFN-γ-CXCL10-CXCR3 inflammatory loop [50]. In our HBZ-Tg mice, however, CXCL10 is not associated with inflammation, since loss of CXCL10 didn’t affect the development of inflammatory diseases. In addition, the upregulation of CXCR3 observed in HBZ-Tg mice was independent of IFN-γ. Therefore CD4^+^ T cells from HBZ-Tg/IFN-γ KO mice still expressed high levels of CXCR3, and could react to its ligand. According to these observations, CXCL10/CXCR3 is unlikely to have strong effects on inflammation induced by HBZ. Indeed, the expression of several other adherent molecules and chemokine receptors such as CCR4, CD29, and CD49d, also showed the same pattern as CXCR3 (Fig 6). Induction of these molecules is mediated by HBZ, but not associated with IFN-γ, suggesting that these molecules might be involved in the inflammation that occurred late in HBZ-Tg/IFN-γ KO mice. Further studies are needed to test this hypothesis.

In conclusion, we showed that IFN-γ, which is secreted by Th1-like cells such as exFoxp3 cells, has important roles in HBZ-mediated inflammation. HBZ increases the number of Tregs in a cell intrinsic manner, and consequently induces IFN-γ in vivo. Importantly, inflammation is closely linked to the development of malignant lymphomas in HBZ-Tg mice. This is the first report showing the relationship between the immunomodulating function of HBZ and oncogenesis that might explain the clinical observations of ATL development in HTLV-1-infected subjects with chronic inflammations.

## Materials and Methods

### Mice

C57BL/6J mice were purchased from CLEA (Tokyo, Japan). Transgenic mice expressing the spliced form of the *HBZ* gene under control of the mouse CD4 promoter have been described previously [13, 16]. B6.129S7-*Ifnγ* ^*tm1Ts*^/J (*Ifnγ* ^*-/-*^) [21] and B6.129S4-*Cxcl10* ^*tm1Adl*^/J (*Cxcl10* ^*-/-*^) [24] mice were purchased from The Jackson Laboratory (CA, USA). Mice used in this study were maintained under SPF conditions unless otherwise specified. GF HBZ-Tg and wild type mice were reconstituted from frozen embryos and reared at the Central Institute for Experimental Animals (Kawasaki, Japan). GF mice aged 18 weeks were transferred to Kyoto University, and analyzed within 24 hours.

### Cell lines

HTLV-1-transformed cell lines (MT-2 and MT-4), ATL cell lines (MT-1, ED, TL-Om1, ATL-43T+, and ATL-55T+) were cultured in RPMI 1640 medium supplemented with 10% fetal bovine serum (FBS) and antibiotics at 37°C under a 5% CO_2_ atmosphere. For IL-2-dependent cell lines (ATL-43T+ and ATL-55T+), recombinant human IL-2 (100 U/ml) was added in the culture media.

### Clinical samples

Peripheral blood mononuclear cells (PBMCs) of ATL patients and healthy donors were collected by Ficoll-Paque PLUS (GE Healthcare). To obtain PHA-stimulated cells, PBMCs were treated with 10μg/ml PHA (Sigma) for 3 days.

### Flow cytometric analysis

The following antibodies were used for flow cytometric analysis of mouse lymphocytes:

Anti-CD3e (145-2C11), CCR5 (C34-3448), IFN-γ (XMG1.2), IL-2 (JES6-5H4), IL-17 (TC11-18H10), CD29 (Ha2/5), CD49d (9C10), and CD162 (2PH1) antibodies were purchased from BD Pharmingen. Anti-CD4 (RM4-5), CD8 (53–6.7), CD44 (IM7), CD62L (MEL-14), CXCR3 (CXCR3-173), CCR4 (2G12), and TNF-α (MP6-XT22) antibodies were from Biolegend. Anti-CD25 (pc61), Foxp3 (FJK-16s), CCR9 (eBioCW-1.2), and IL-4 (11B11) antibodies were from eBioscience. Anti-CCR10 antibody (248918) was purchased from R&D systems. In order to stain cytokines, splenocytes were stimulated with 50ng/mL PMA (Nakarai Tesque), 1μg/mL ionomycin (Nakarai Tesque) and a protein transport inhibitor, BD Golgi plug (BD Pharmingen) for 4 hours before harvesting cells. After cell surface staining, cells were fixed and permeabilized with Fixation/Permeabilization working solution (eBioscience) and intracellular antigens were stained. Flow cytometric analysis was carried out using a FACS Verse with FACSuite software (BD Biosciences) and Flow Jo (FlowJo, LLC).

### Histological analysis

Mouse tissues were fixed in 10% formalin in phosphate buffer (Nakarai Tesque) and then embedded in paraffin. Hematoxylin and eosin staining was performed according to standard procedures. Images were captured using a Provis AX80 microscope (Olympus) equipped an OLYMPUS DP70 digital camera, and detected using a DP manager system (Olympus).

### Migration assay

Mouse CD4^+^ T cells were isolated from splenocytes by CD4 T lymphocyte enrichment Set-DM (BD Biosciences) and resuspended in RPMI containing 0.1% BSA. To evaluate migration activity, a Transwell insert (3.0um) (CORNING) was used. The lower chamber was filled with chemotaxis medium containing mouse recombinant CXCL10 (R&D systems). One million cells were added into the upper chamber. The chamber was incubated for 1 hour at 37C and 5% CO_2_. Cells that migrated towards CXCL10 were counted using Flow cytometry.

### Microarray analysis

CD4^+^ T cells were isolated from WT, HBZ-Tg, IFN-γ KO and HBZ-Tg/IFN-γ KO mice as described above and lysed in TRIzol (Life Technologies). Total RNAs were extracted from these lysates with Direct-zol RNA MiniPrep (Zymo Research). RNA quality was checked using Agilent 2100 Bioanalyzer (Agilent Technologies). Microarray experiments were carried out with SurePrint G3 Mouse GE 8x60K (Agilent Technologies) according to manufacturer’s instructions. Data was analyzed with GeneSpring GX software (Agilent Technologies).

### Quantitative RT-PCR

Splenocytes harvested from WT, HBZ-Tg, IFN-γ KO, and HBZ-Tg/IFN-γ KO mice and human PBMCs obtained from ATL patients and healthy donors were lysed with TRIzol reagent, and RNA was extracted as described above. cDNAs were synthesized from 1μg of total RNAs using random primers and SuperScript III Reverse Transcriptase (Life Technologies). The expression levels of candidate genes were quantified by the StepOnePlus real time PCR system (Life Technologies) using FastStart Universal SYBR Green Master (Roche). Relative expression levels of each gene were calculated by the delta delta Ct method [51]. The sequences of primers used in this study are listed in S2 Table. Human *NRXN3* was quantified using Taqman Gene Expression Assays (Applied Biosystems, Hs01028186_m1).

### Ethics statement

Animal experiments were performed in strict accordance with the Japanese animal welfare bodies (Law No. 105 dated 19 October 1973 modified on 2 June 2006), and the Regulation on Animal Experimentation at Kyoto University. The protocol was approved by the Institutional Animal Research Committee of Kyoto University (Permit numbers are D13-02, D14-02, and D15-02). Experiments using clinical samples were conducted according to the principles expressed in the Declaration of Helsinki, and approved by the Institutional Review Board of Kyoto University (Permit numbers are G310 and G204). ATL patients provided written informed consent for the collection of samples and subsequent analysis.

## Supporting Information

S1 Fig Genotyping of HBZ-Tg/IFN-γ KO mice. Genotyping of WT, HBZ-Tg, IFN-γ KO, and HBZ-Tg/IFN-γ KO mice was carried out by PCR.
(PPTX)

S2 Fig Representative dot plots of T-cell subsets in HBZ-Tg and HBZ-Tg/IFN-γ KO mice. (A) Splenocytes were harvested from WT, HBZ-Tg, IFN-γ KO, and HBZ-Tg/IFN-γ KO mice at 24-week old. Cells were stained with anti-CD4, anti-Foxp3, anti-CD25 antibodies for detection of regulatory T cells, and anti-CD44, anti-CD62L antibodies for effector/memory CD4^+^ T cells. Representative results are shown. (B) Cytokine production in CD4^+^ T cells was evaluated. Splenocytes were stimulated with PMA/ionomycin in the presence of protein transport inhibitor for 4 hours, stained with specific antibodies, and analyzed by flow cytometry. Representative results are shown.
(PPTX)

S3 Fig Inflammatory phenotypes of SPF HBZ-Tg mice. (A) Splenocytes were harvested from 18-week-old SPF HBZ-Tg or SPF WT littermates. The percentages of Tregs and effector/memory CD4^+^ T cells were evaluated. Representative results of the dot plots and a summarized table are shown. (B) Cytokine production in CD4^+^ T cells was evaluated. Splenocytes were stimulated with PMA/ionomycin in the presence of protein transport inhibitor for 4 hours, stained with specific antibodies, and analyzed by flow cytometry. Representative results of the dot plots and a summarized table are shown.
(PPTX)

S1 Table Quantification of the candidate genes in HTLV-1-infected cell lines. Each value was calculated by the delta delta Ct method using a resting HD sample as reference. N.D.: not detected.
(DOCX)

S2 Table Primers for quantitative RT-PCR. (DOCX)
