# Correction: Interferon-γ Promotes Inflammation and Development of T-Cell Lymphoma in HTLV-1 bZIP Factor Transgenic Mice

**DOI:** 10.1371/journal.ppat.1005214

**Published:** 2015-10-09

**Authors:** Yu Mitagami, Jun-ichirou Yasunaga, Haruka Kinosada, Koichi Ohshima, Masao Matsuoka

There is funding information omitted from the Funding Statement in this article. The corrected Funding Statement is provided here:

This study was supported by grants from the Ministry of Education, Science, Sports, and Culture of Japan to M.M. (22114003) and J.Y. (26460554), and a grant from the Takeda Science Foundation (J.Y.). This study was performed as a research program of the Platform for Dynamic Approaches to Living System from the Ministry of Education, Culture, Sports, Science and Technology, Japan (Y.M.). This study was also supported in part by the JSPS Core-to-Core Program A, Advanced Research Networks, and the Joint Usage/Research Center program of the Institute for Virus Research, Kyoto University.
