# Supplementary figures and images for: Interferon-γ Promotes Inflammation and Development of T-Cell Lymphoma in HTLV-1 bZIP Factor Transgenic Mice
Source: PLoS Pathog. 2015 Aug 21;11(8):e1005120. doi: 10.1371/journal.ppat.1005120 (PMC4546626; doi:10.1371/journal.ppat.1005120)

## Slide 1
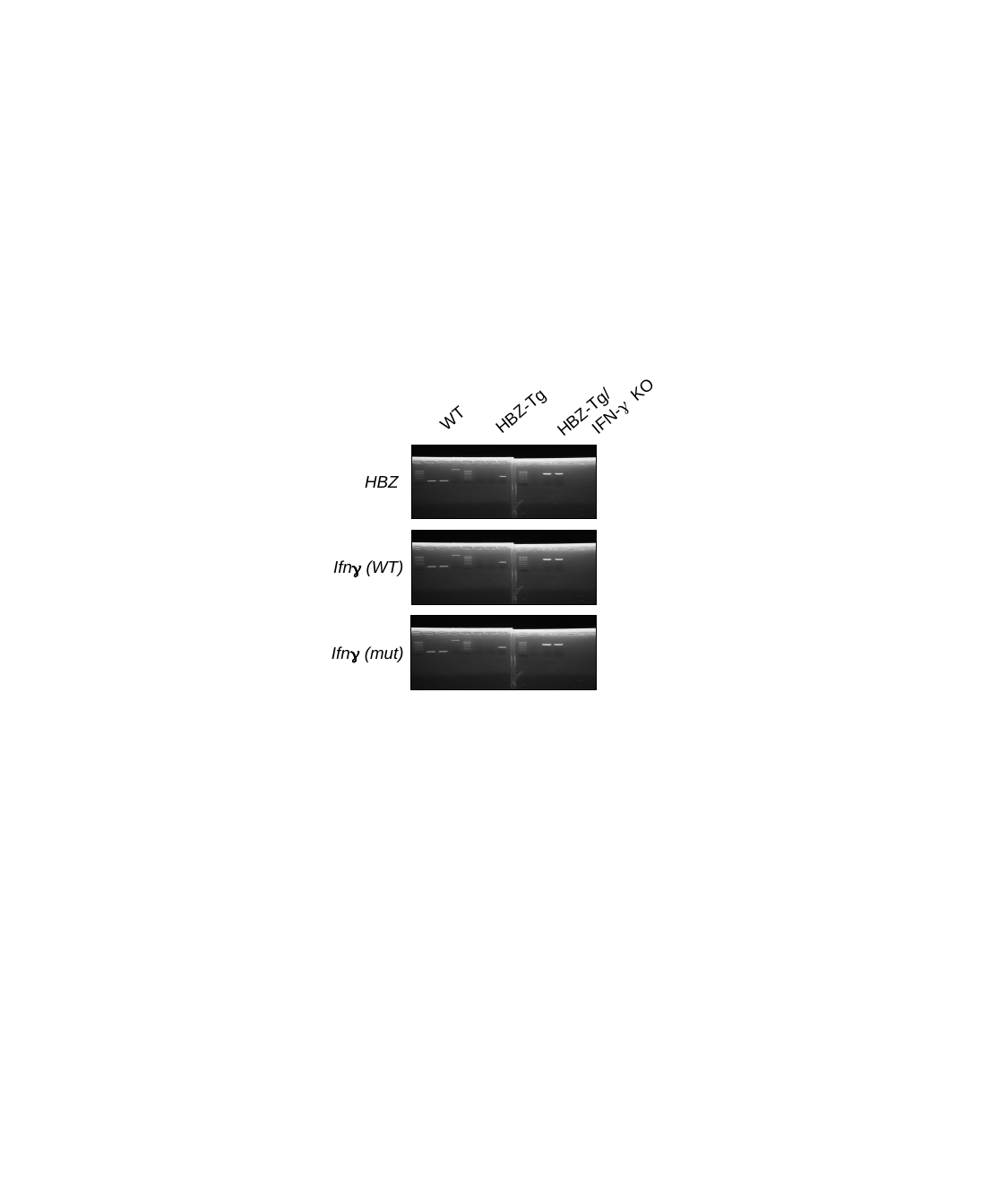

HBZ-Tg/
 IFN-g KO
HBZ-Tg
WT
HBZ
Ifng (WT)
Ifng (mut)

Supplement: S1 Fig — Genotyping of WT, HBZ-Tg, IFN-γ KO, and HBZ-Tg/IFN-γ KO mice was carried out by PCR. (PPTX) [file ppat.1005120.s001.pptx]
