# Supplementary material for: Interferon-γ Promotes Inflammation and Development of T-Cell Lymphoma in HTLV-1 bZIP Factor Transgenic Mice
Source: PLoS Pathog. 2015 Aug 21;11(8):e1005120. doi: 10.1371/journal.ppat.1005120 (PMC4546626; doi:10.1371/journal.ppat.1005120)
Supplement: S2 Fig — (A) Splenocytes were harvested from WT, HBZ-Tg, IFN-γ KO, and HBZ-Tg/IFN-γ KO mice at 24-week old. Cells were stained with anti-CD4, anti-Foxp3, anti-CD25 antibodies for detection of regulatory T cells, and anti-CD44, anti-CD62L antibodies for effector/memory CD4+ T cells. Representative results are shown. (B) Cytokine production in CD4+ T cells was evaluated. Splenocytes were stimulated with PMA/ionomycin in the presence of protein transport inhibitor for 4 hours, stained with specific antibodies, and analyzed by flow cytometry. Representative results are shown. (PPTX) [file ppat.1005120.s002.pptx]

## Slide 1
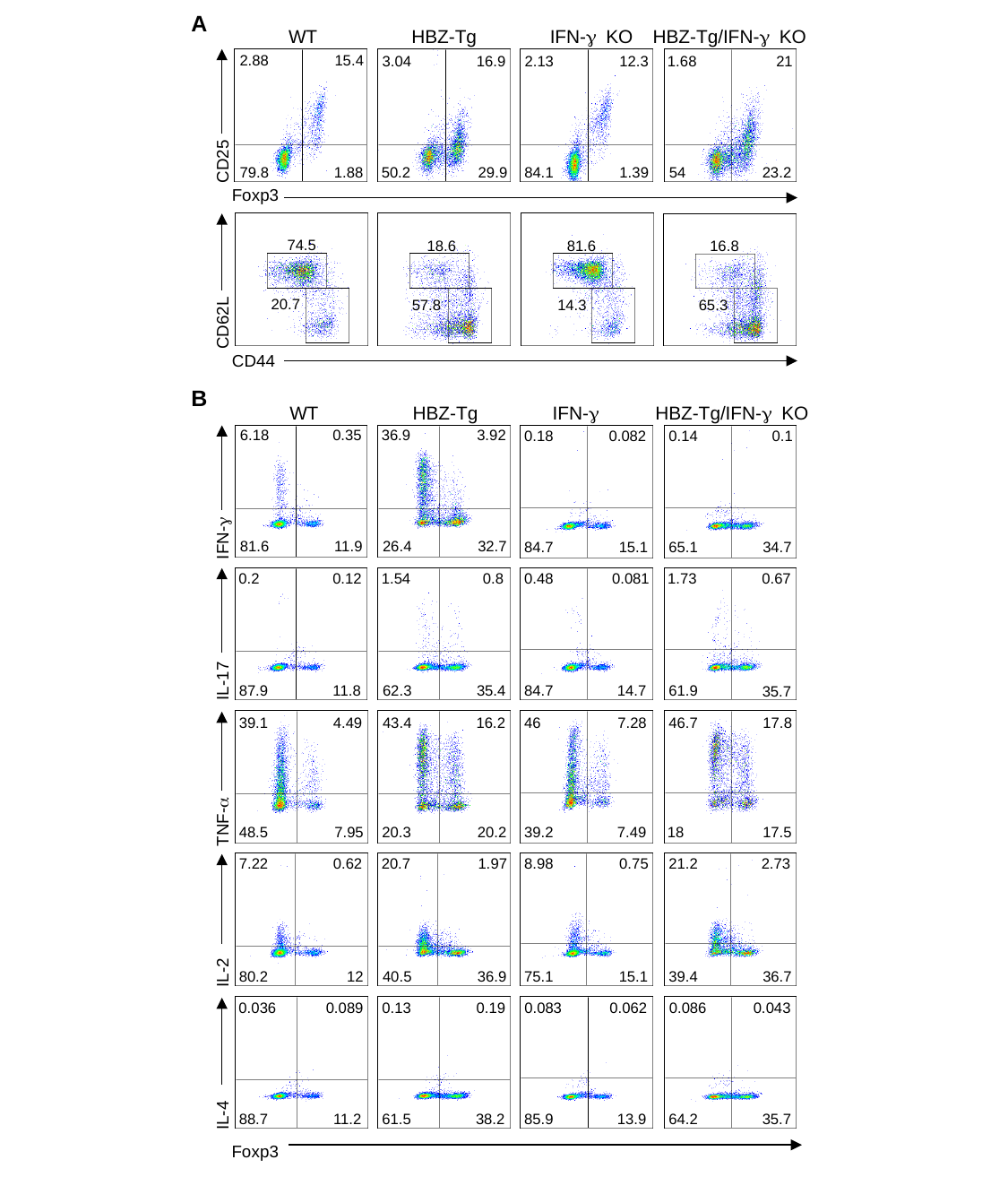

A
WT
HBZ-Tg
IFN-g KO
HBZ-Tg/IFN-g KO
2.88
15.4
3.04
16.9
2.13
12.3
1.68
21
CD25
79.8
1.88
50.2
29.9
84.1
1.39
54
23.2
Foxp3
74.5
18.6
81.6
16.8
20.7
57.8
14.3
65.3
CD62L
CD44
B
HBZ-Tg/IFN-g KO
WT
HBZ-Tg
IFN-g KO
6.18
0.35
36.9
3.92
0.18
0.082
0.14
0.1
IFN-g
81.6
11.9
26.4
32.7
84.7
15.1
65.1
34.7
0.2
0.12
1.54
0.8
0.48
0.081
1.73
0.67
IL-17
87.9
11.8
62.3
35.4
84.7
14.7
61.9
35.7
39.1
4.49
43.4
16.2
46
7.28
46.7
17.8
TNF-a
48.5
7.95
20.3
20.2
39.2
7.49
18
17.5
7.22
0.62
20.7
1.97
8.98
0.75
21.2
2.73
IL-2
80.2
12
40.5
36.9
75.1
15.1
39.4
36.7
0.036
0.089
0.13
0.19
0.083
0.062
0.086
0.043
IL-4
88.7
11.2
61.5
38.2
85.9
13.9
64.2
35.7
Foxp3
