# Supplementary material for: Interferon-γ Promotes Inflammation and Development of T-Cell Lymphoma in HTLV-1 bZIP Factor Transgenic Mice
Source: PLoS Pathog. 2015 Aug 21;11(8):e1005120. doi: 10.1371/journal.ppat.1005120 (PMC4546626; doi:10.1371/journal.ppat.1005120)
Supplement: S3 Fig — (A) Splenocytes were harvested from 18-week-old SPF HBZ-Tg or SPF WT littermates. The percentages of Tregs and effector/memory CD4+ T cells were evaluated. Representative results of the dot plots and a summarized table are shown. (B) Cytokine production in CD4+ T cells was evaluated. Splenocytes were stimulated with PMA/ionomycin in the presence of protein transport inhibitor for 4 hours, stained with specific antibodies, and analyzed by flow cytometry. Representative results of the dot plots and a summarized table are shown. (PPTX) [file ppat.1005120.s003.pptx]

## Slide 1
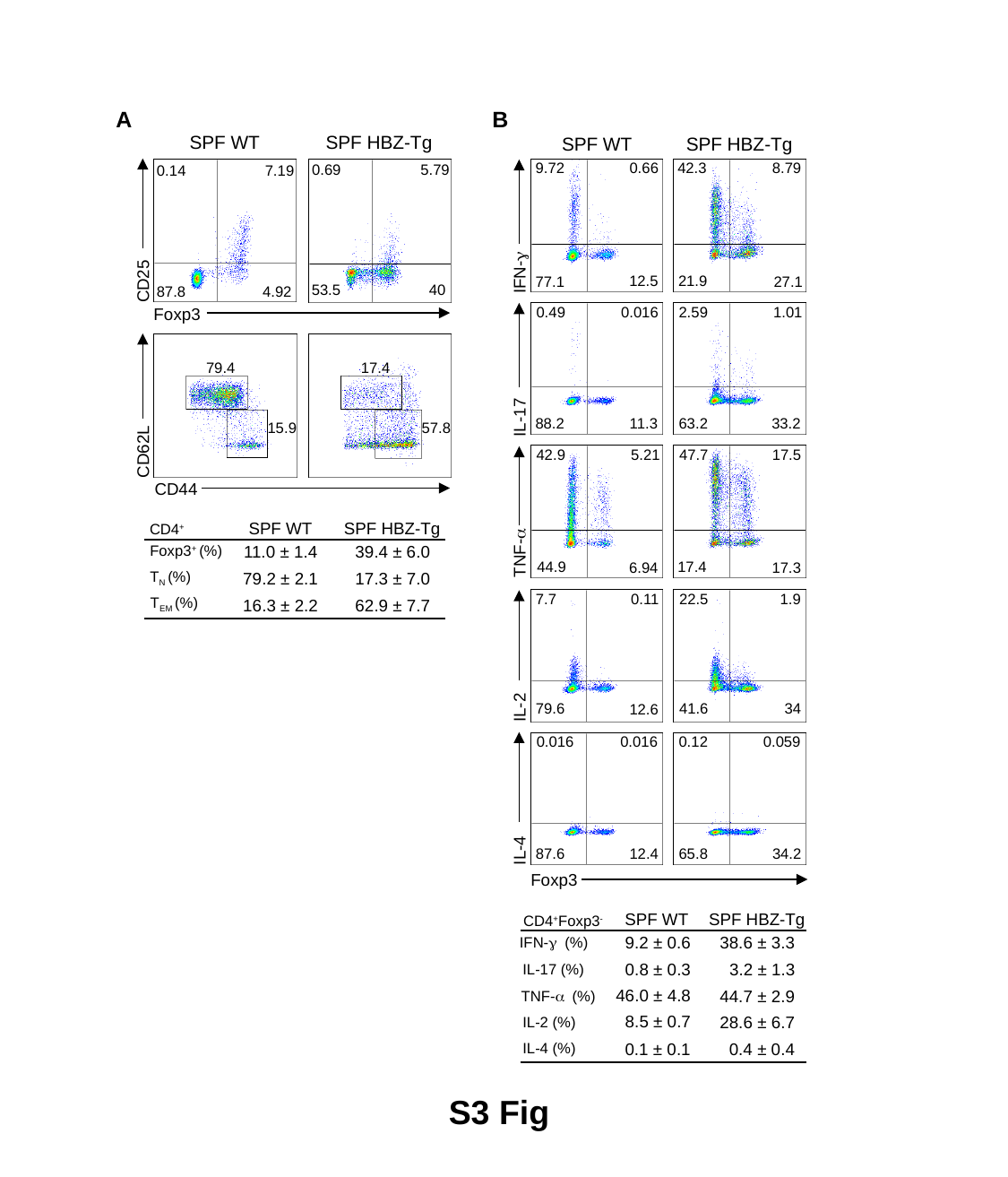

A
B
SPF WT
SPF HBZ-Tg
SPF WT
SPF HBZ-Tg
9.72
0.66
42.3
8.79
0.69
5.79
0.14
7.19
IFN-g
CD25
21.9
12.5
77.1
27.1
53.5
40
87.8
4.92
0.49
0.016
2.59
1.01
Foxp3
17.4
79.4
IL-17
11.3
88.2
33.2
63.2
57.8
15.9
CD62L
42.9
5.21
47.7
17.5
CD44
SPF WT
SPF HBZ-Tg
CD4+
Foxp3+ (%)
11.0 ± 1.4
39.4 ± 6.0
TNF-a
44.9
17.4
17.3
6.94
TN (%)
79.2 ± 2.1
17.3 ± 7.0
7.7
0.11
22.5
1.9
TEM (%)
16.3 ± 2.2
62.9 ± 7.7
IL-2
79.6
34
41.6
12.6
0.016
0.016
0.12
0.059
IL-4
12.4
34.2
87.6
65.8
Foxp3
SPF WT
SPF HBZ-Tg
CD4+Foxp3-
9.2 ± 0.6
38.6 ± 3.3
IFN-g (%)
0.8 ± 0.3
3.2 ± 1.3
IL-17 (%)
46.0 ± 4.8
44.7 ± 2.9
TNF-a (%)
8.5 ± 0.7
28.6 ± 6.7
IL-2 (%)
0.4 ± 0.4
IL-4 (%)
0.1 ± 0.1
S3 Fig
