# Supplementary material for: Interferon-γ Promotes Inflammation and Development of T-Cell Lymphoma in HTLV-1 bZIP Factor Transgenic Mice
Source: PLoS Pathog. 2015 Aug 21;11(8):e1005120. doi: 10.1371/journal.ppat.1005120 (PMC4546626; doi:10.1371/journal.ppat.1005120)
Supplement: S1 Table — Each value was calculated by the delta delta Ct method using a resting HD sample as reference. N.D.: not detected. (DOCX) [file ppat.1005120.s004.docx]

**S1 Table. Quantification of the candidate genes in HTLV-1-infected cell lines**

|  | MT-1 | MT-2 | MT-4 | TL-Om1 | ED | ATL-43T+ | ATL-55T+ |
| --- | --- | --- | --- | --- | --- | --- | --- |
| *NEO1* | 22.0 | 8.5 | 49.0 | 16.8 | 23.6 | 18.9 | 4.4 |
| *IL1F9* | 55.0 | 17.3 | 3505.0 | 14.8 | N.D. | 22.4 | 4.7 |
| *FGFR4* | 53.8 | 7.2 | 34.0 | 1.2 | 1.6 | 7.3 | N.D. |
| *HIP1* | 9.6 | 8.1 | 24.5 | 19.5 | 1.7 | 10.6 | 33.0 |
| *IKZF2* | 14.9 | 0.1 | 0.1 | 11.9 | 0.8 | 22.0 | 38.4 |
| *NRXN3* | N.D. | 153.3 | N.D. | N.D. | 5423.3 | N.D. | 25.5 |
