# Supplementary material for: Interferon-γ Promotes Inflammation and Development of T-Cell Lymphoma in HTLV-1 bZIP Factor Transgenic Mice
Source: PLoS Pathog. 2015 Aug 21;11(8):e1005120. doi: 10.1371/journal.ppat.1005120 (PMC4546626; doi:10.1371/journal.ppat.1005120)
Supplement: S2 Table — (DOCX) [file ppat.1005120.s005.docx]

**S2 Table. Primers for quantitative RT-PCR**

| spieces | gene |  | sequence |
| --- | --- | --- | --- |
| mouse | *Fgfr4* | F | 5'-AAATGTGCTGGTGACCGAGG-3' |
|  |  | R | 5'-GTACACGCGGTCGAACAATG-3' |
|  | *Hip1* | F | 5'-CGAGGAGCTGATGGTGTGTTC-3' |
|  |  | R | 5'-AAATGGTTGAGGCCACCACA-3' |
|  | *Ikzf2* | F | 5'-CAAGGAACGCTGTCACAACT-3' |
|  |  | R | 5'-TCTTCCATAGGCGGTACATGG-3' |
|  | *Il1f9* | F | 5'-AGAGTAACCCCAGTCAGCGTG-3' |
|  |  | R | 5'-AGGGTGGTGGTACAAATCCAA-3' |
|  | *Neo1* | F | 5'-CAGTGTGTAGCCACTGTGGATA-3' |
|  |  | R | 5'-AAGGTTCTGGTTGGCTGGTAAA-3' |
|  | *Nrxn3* | F | 5'-ATGTCTACCACCGTCATGGA-3' |
|  |  | R | 5'-ACAAGATCATCTGACGTGGG-3' |
|  | *Rn18s* | F | 5'-GTAACCCGTTGAACCCCATT-3' |
|  |  | R | 5'-CCATCCAATCGGTAGTAGCG-3' |
| human | *FGFR4* | F | 5'-GGGAGGGACATCGGATGGA-3' |
|  |  | R | 5'-CCACCAGCTGCTTGAAGGTA-3' |
|  | *HIP1* | F | 5'-AGGCCATTAATACGCAGGAAGT-3' |
|  |  | R | 5'-CAGAAGGTCTGTGCCCCTTT-3' |
|  | *IKZF2* | F | 5'-TCATAGAGAAGCTCACGGGGA-3' |
|  |  | R | 5'-TGGGTAGCTGAATCGCATGA-3' |
|  | *IL1F9* | F | 5'-ACAGCCCACATTGCAGCTAA-3' |
|  |  | R | 5'-CCAGTCTTGGCACGGTAGAA-3' |
|  | *NEO1* | F | 5'-GGCCACTGTTGAGAGTCTTG-3' |
|  |  | R | 5'-GGTTCTGGTTGGCTGGTAAA-3' |
|  | *RNA18s5* | F | 5'-AACCCGTTGAACCCCATT-3' |
|  |  | R | 5'-CCATCCAATCGGTAGTAGCG-3' |
